# Reduced presynaptic vesicle stores mediate cellular and network plasticity defects in an early-stage mouse model of Alzheimer’s disease

**DOI:** 10.1186/s13024-019-0307-7

**Published:** 2019-01-22

**Authors:** Shreaya Chakroborty, Evan S. Hill, Daniel T. Christian, Rosalind Helfrich, Shannon Riley, Corinne Schneider, Nicolas Kapecki, Sarah Mustaly-Kalimi, Figen A. Seiler, Daniel A. Peterson, Anthony R. West, Barbara M. Vertel, William N. Frost, Grace E. Stutzmann

**Affiliations:** 1 Department of Neuroscience, The Chicago Medical School; The Center for Neurodegenerative Disease and Therapeutics, Rosalind Franklin University of Medicine and Science, 3333 Green Bay Rd, North Chicago, IL 60064 USA; 2 Department of Cell Biology and Anatomy, The Chicago Medical School; Center for Brain Function and Repair, Rosalind Franklin University of Medicine and Science, 3333 Green Bay Rd, North Chicago, IL 60064 USA; 3 Electron Microscopy Center, RFUMS, North Chicago, IL 60064 USA

**Keywords:** Synaptic, Hippocampus, Short-term plasticity, Synaptic vesicles, Calcium, Ryanodine receptor, Patch clamp, 2-photon imaging, Electron microscopy, Network imaging, Mouse model, Spines, Alzheimer’s disease

## Abstract

**Background:**

Identifying effective strategies to prevent memory loss in AD has eluded researchers to date, and likely reflects insufficient understanding of early pathogenic mechanisms directly affecting memory encoding. As synaptic loss best correlates with memory loss in AD, refocusing efforts to identify factors driving synaptic impairments may provide the critical insight needed to advance the field. In this study, we reveal a previously undescribed cascade of events underlying pre and postsynaptic hippocampal signaling deficits linked to cognitive decline in AD. These profound alterations in synaptic plasticity, intracellular Ca^2+^ signaling, and network propagation are observed in 3–4 month old 3xTg-AD mice, an age which does not yet show overt histopathology or major behavioral deficits.

**Methods:**

In this study, we examined hippocampal synaptic structure and function from the ultrastructural level to the network level using a range of techniques including electron microscopy (EM), patch clamp and field potential electrophysiology, synaptic immunolabeling, spine morphology analyses, 2-photon Ca^2+^ imaging, and voltage-sensitive dye-based imaging of hippocampal network function in 3–4 month old 3xTg-AD and age/background strain control mice.

**Results:**

In 3xTg-AD mice, short-term plasticity at the CA1-CA3 Schaffer collateral synapse is profoundly impaired; this has broader implications for setting long-term plasticity thresholds. Alterations in spontaneous vesicle release and paired-pulse facilitation implicated presynaptic signaling abnormalities, and EM analysis revealed a reduction in the ready-releasable and reserve pools of presynaptic vesicles in CA3 terminals; this is an entirely new finding in the field. Concurrently, increased synaptically-evoked Ca^2+^ in CA1 spines triggered by LTP-inducing tetani is further enhanced during PTP and E-LTP epochs, and is accompanied by impaired synaptic structure and spine morphology. Notably, vesicle stores, synaptic structure and short-term plasticity are restored by normalizing intracellular Ca^2+^ signaling in the AD mice.

**Conclusions:**

These findings suggest the Ca^2+^ dyshomeostasis within synaptic compartments has an early and fundamental role in driving synaptic pathophysiology in early stages of AD, and may thus reflect a foundational disease feature driving later cognitive impairment. The overall significance is the identification of previously unidentified defects in pre and postsynaptic compartments affecting synaptic vesicle stores, synaptic plasticity, and network propagation, which directly impact memory encoding.

**Electronic supplementary material:**

The online version of this article (10.1186/s13024-019-0307-7) contains supplementary material, which is available to authorized users.

## Background

Research on the etiology of Alzheimer’s disease (AD) and cause of memory decay in AD has long focused on advanced histological hallmarks of aging and disease such as amyloid and tau aggregations. Yet, increasing evidence indicates that defects in synaptic structure and function play an earlier role in AD-associated memory decline [1–4]. This makes intuitive sense, as synapses are the initiating sites of most forms of memory encoding. Altered hippocampal long-term plasticity (LTP), one of the cellular correlates of learning and memory, has been well characterized across many AD mouse models at later disease stages, often concurrent with overt amyloid histopathology and memory impairments [5, 6]. However, more subtle and insidious pathophysiological mechanisms are evident prior to these observations. To date, these remain insufficiently investigated and thus poorly understood. For example, hippocampal short-term plasticity (STP), which gates information processing and working memory, is often overlooked as a significant contributor to memory deficits in dementia. Post-tetanic potentiation (PTP), a form of presynaptic STP, is essential for setting LTP thresholds, synchronizing network propagation, and enabling short-term memory [7–9]. Many forms of STP are Ca^2+^ dependent [10] and thus, the early Ca^2+^ signaling abnormalities in AD can alter synaptic signaling and hippocampal network propagation. This can manifest as working memory deficits, consistent with those observed in 3-month old 3xTg-AD mice [11, 12].

While a substantial body of data characterizing long-term plasticity abnormalities in AD models exists, further investigation into upstream mechanisms is needed to understand causes of AD-linked memory loss. One of the earliest observed neuronal signaling alterations that directly associates with AD features and risk factors, such as amyloid and tau pathology, synaptic deficits, oxidative stress, and cell death, is intracellular Ca^2+^ dysregulation [2, 13–15]. Increased Ca^2+^ release through IP_3_R and RyR-localized ER channels occurs as early as 2 weeks of age in AD mouse models [16, 17], is observed in cells from FAD patients [18–20], and is 2–10 fold greater than control responses within dendrites and spines [21, 22]. Synaptic compartments are particularly vulnerable to dysregulated RyR-Ca^2+^ signaling, and can result in impaired dendritic structure and function [23–25], altered presynaptic vesicle release properties [26], and synaptic plasticity deficits, all of which play key roles in memory processes [27–31]. A deeper investigation into these proximal components, such as the state of presynaptic neurotransmitter vesicle stores and the Ca^2+^ microenvironment is still needed. For example, increased spontaneous vesicle release activity occurs with PTP and derives from increased Ca^2+^ within terminals [32–34]. Alterations in these variables compromise the strength and fidelity of PTP, and thus interfere with LTP gating within individual synapses and across networks.

Here, we conducted a detailed study into early structural and functional hippocampal synaptic abnormalities, from the ultrastructural to network levels, in 3 month old AD mice to pinpoint early mechanisms of memory loss. We reveal a maladaptive cascade in 3xTg-AD mice that includes impaired STP, reduced vesicle content in presynaptic active zones, aberrant meta-plasticity in dendritic Ca^2+^ responses, and spatially and temporally blunted network plasticity in the stratum oriens (SO) CA1 subfield. Our findings expose profound changes that emerge prior to the overt proteinopathy and associative memory deficits that define AD, and offer new perspectives on mechanisms to prioritize for AD therapeutics.

## Methods

### Mouse models

Three to four-month old male and female 3xTg-AD mice (PS1_M146V_, APP_SWE_, Tau_P301L_; [6] and age-matched background strain controls (C57bl6/J9) were used in this study. The 3xTg-AD mouse model was chosen for several reasons. For example, this mouse model has been extensively used and characterized for over 15 years, with over 600 PubMed citations. Furthermore, we have observed a highly consistent phenotype over time which likely reflects the PS1 KI gene [6, 17, 22], and deficits in PS function and the associated Ca^2+^ dysregulations are applicable to both familial and sporadic AD [35]. While the field has noted a delay in the onset of amyloid and tau histopathology, we have obtained mice from the original cohort that maintains the histopathology pattern as described in [4].

### Hippocampal slice preparation

Mice were deeply anesthetized with isoflurane, decapitated, and the brains dissected into ice-cold sucrose cutting solution (SCS, in mM: 200 sucrose, 1.5 KCl, 0.5 CaCl_2_, 4.0 MgCl_2_, 1.0 KH_2_PO_4_, 25 NaHCO_3_, 10 Na-ascorbate and 20 dextrose, equilibrated with 95% O_2_/5% CO_2_.) Transverse hippocampal slices (300 μm for whole-cell patch clamp recordings and 400 μm for VSD imaging) were prepared on a Camden Instruments vibratome with the chamber filled with ice-cold SCS and then transferred to a holding chamber where they were maintained at 30 °C in standard artificial cerebrospinal fluid (aCSF, in mM: 130 NaCl, 2.5 KCl, 2.0 CaCl_2_, 1.2 MgSO_4_, 1.25 KH_2_PO_4_, 25 NaHCO_3_ and 10 dextrose) bubbled continuously with 95% O_2_/5% CO_2_, pH 7.3–7.4, for at least 1 h before use.

### Whole cell patch clamp recording

Hippocampal brain slices (300 μm) were placed in a perfusion chamber mounted on a movable stage assembly on an upright microscope (BX50WI; Olympus Optical) and superfused at 2 ml/min with aCSF equilibrated with 95% O_2_/5% CO_2_ at room temperature (27 °C). Patch pipettes (4–5 MΩ) pulled from borosilicate glass tubing were filled with intracellular solution (in mM: 135 K-gluconate, 2 MgCl_2_, 4 Na-ATP, 0.4 Na-GTP, 10 Na-phosphocreatine and 10 HEPES, pH adjusted to 7.3 with KOH) and 100 μM bis-fura-2. Hippocampal CA1 pyramidal neurons were identified visually via IR-DIC optics, and electrophysiologically by their membrane properties and spike frequency accommodation. Membrane potentials and evoked responses were obtained in current-clamp mode using pClamp 10 software acquired at 10 kHz with a Digidata 1322 A-D converter and MultiClamp 700B amplifier. The current-voltage relationship was assessed by measuring voltage responses to constant current pulses of 500 ms duration and varying amplitudes from − 200 to + 200 pA in steps of 20 pA. Spontaneous postsynaptic potentials (sEPSPs) were recorded for 1 min. Synaptic responses were evoked by stimulating the Schaffer collateral/commissural pathway with a monopolar stimulating electrode. Input/Output (I/O) curves were generated using stimulus intensities from 0 to 500 pA in steps of 50 pA. Baseline stimulus intensity was determined from I/O curves as the stimulus intensity evoking a 30% of maximal response. Plasticity was induced by a tetanus consisting of two 100 Hz trains, 10 s apart. Paired pulse facilitation (PPF) was assessed before and after tetanus using interstimulus intervals of 25, 50 and 200 ms. Five successive responses were recorded for each interstimulus interval at 0.05 Hz. To ensure slice viability and stable recording conditions within and between slices, membrane input resistance, membrane potential, AP waveform and amplitude, and electrode access resistance were continually monitored throughout the experiment; cells were used for recording only if the access resistance was maintained < 10 MΩ, and all other parameters remained stable.

### Ca^2+^ imaging

Imaging of fluorescent Ca^2+^ signals was performed in acute hippocampal brain slices using a custom-made video-rate multiphoton imaging system [36]. Individual CA1 pyramidal neurons were filled with the Ca^2+^ indicator bis-fura-2 via a patch pipette and Ca^2+^ responses imaged from dendrites and dendritic spines. Laser excitation was provided by 80 MHz trains of ultra-short (100 fs) pulses at 780 nm from a titanium/sapphire laser (Mai Tai Broadband, Spectra-Physics). The laser beam was scanned by paired galvanometers to provide a full-frame scan rate of 30 Hz. The scanned beam was focused onto the tissue through an Olympus 40X water immersion objective (NA 0.8). Emitted fluorescence light was detected by a wide-field photomultiplier (R5929, Hamamatsu) and captured by frame-grabber software VideoSavant 5.0 (IO Industries). Imaging was synced with electrophysiological protocols through Digidata 1322 A-D board controlled by pClamp 10 software. 30 Hz trains were administered at baseline stimulus intensity. Images were analyzed offline with MetaMorph v7.8.6.0 software.

### Voltage sensitive dye imaging

Hippocampal slices (400 μm) were used for imaging experiments. Slices were equilibrated with 95% O_2_/5% CO_2_ in aCSF, as described above. The VSD RH 155 dye was bath applied at a concentration of 0.03 mg/ml (in aCSF) for 1.5 to 2 h. Imaging was performed in dye-free aCSF. Schaffer collateral fibers were stimulated with a monopolar stimulating electrode using the stimulus intensity that evoked 50% of the maximal optical response in CA1 (200–400 μA, 100 μs). Synaptic plasticity was induced at baseline intensity by a tetanus consisting of two 100 Hz trains, 10 s apart. Optical responses were acquired with a RedShirtImaging CMOS camera with 128 × 128 pixels and a sampling rate of 1250 Hz. Optical data were band-pass filtered (125 Hz LP, 1 Hz HP), and signals were divided by the resting light intensity at each pixel to yield measurements of DI/I using Neuroplex software. For spread analysis, a 6 × 6 binning was used to increase the signal-to-noise ratio of the optical responses. Data were analyzed offline using a custom MATLAB script to measure the amplitude of each trace, to identify the bin with the maximal response and calculate the percentage of bins above 50% of the maximal response. The range of color bar was set to strongest responding pixel or bin and the cut off for the color bar was set at 50%. For duration analysis the time from 10 to 10% of the peak (rise and fall) of the optical response was calculated. Data were assessed for significance using repeated measures two-way ANOVAs with Student-Newman-Keuls post hoc analysis in SigmaPlot 11.

### Transmission Electron microscopy

Mice were deeply anesthetized with halothane, and transcardially perfused with ice-cold modified Karnovsky’s 2% paraformaldehyde and 2.5% glutaraldehyde, followed by immersion fixation in the same fixative for 7 days. Hippocampal sections of 3xTg-AD mice and NonTg controls were blocked in 200 μm sections trimmed to the CA1 SR subfield, followed by further fixation and dehydration as described previously [37]. Sections were then flat embedded in epoxy resin between two sheets of Aclar Fluorohalocarbon film [38]. After polymerization, sections of the CA1 SR subfield were cut using a Leica EMUC6 ultramicrotome, stained with uranyl acetate and lead citrate and imaged using a JEOL JEM-1230 transmission electron microscope equipped with a Hamamatsu ORCA-HR CCD Camera (AMT XR-60 imaging system). Excitatory asymmetric synapses within the CA1 SR subfield were focused upon, as these constitute the major glutamatergic Schaffer collateral inputs from the CA3 subfield [39, 40], and this approach allows us to align the ultrastructural data with our electrophysiological and synaptic Ca^2+^ imaging data. Electron micrographs at a magnification of 30,000X were analyzed using ImageJ software to measure post synaptic density length and number of vesicles in the readily releasable, reserve and resting pools in each group, as described [41]. Experimenters were blind to mouse strain.

### Dantrolene treatment and dose schedule

A nanocrystal formulation of dantrolene, Ryanodex (Lyotropic Therapeutics Inc.) was administered intraperitoneally at 10 mg/kg [42] to 3xTg-AD and NonTg mice. Mice were injected daily for 4 weeks starting at 2 months of age. Control 3xTg-AD and NonTg mice were administered 0.9% saline daily.

### Dendritic spine imaging

Mice were transcardially perfused with cold 1% paraformaldehyde in phosphate-buffered saline (PBS, pH 7.4) for 1 min, followed by cold 4% paraformaldehyde with 0.125% glutaraldehyde in PBS for 12 min. Brains were extracted and post-fixed in the same fixative for 12 h. Coronal hippocampal sections 200 μm thick were cut from the fixed brains. Hippocampal sections were floated in 1X PBS in a glass petri dish with a clear glass slide glued to the bottom. The section was secured on the slide with a nitrocellulose membrane and metal washers. The hippocampal CA1 subfield was visualized through a cut out segment of the nitrocellulose membrane. Sharp electrodes were pulled from glass capillaries and filled with a 5% (100 mM) Lucifer Yellow (LY) solution. The solution was visualized at 4X magnification with ultraviolet illumination. Electrodes were placed into the tissue and systematically moved on a diagonal through the thickness of the brain section. When the membrane of a CA1 neuron was pierced, the LY solution was allowed to passively diffuse into the soma and proximal dendritic arbor, allowing visualization of the neuron. Injection of a constant negative current (1–5 nA) was then delivered to allow robust filling of the entire dendritic arbor [43, 44]. Electric current was ceased at the first sign of LY leak from the injection site (typically 1–3 min) and the electrode removed from the soma. Single electrodes were re-used to fill multiple cells throughout the CA1 subfield. The filled neurons were spaced apart so that there was no overlap of filled dendritic arbors. Sections with LY-filled neurons were mounted in Vectashield mounting medium (Vector Laboratories) on slides with a 120 μm spacer (Electron Microscopy Sciences), coverslipped and sealed for imaging. All slides were stored at 4 °C.

### Immunohistochemistry

(see [28] for further details.) Mice were deeply anesthetized with urethane (1.5 g/kg) and then transcardially perfused with ice-cold buffered saline followed by 4% paraformaldehyde. Brains were extracted and post-fixed for 12–24 h, then cut on a Leica SM 2010R sliding microtome at 40 μm thickness. Free-floating hippocampal sections were washed with TBS + 0.3% Triton-X (3 × 5 minutes). Sections were blocked with 0.3% Triton-X + 10% Goat serum for 1 h and then incubated in primary antibody (PSD-95,1:500, Cell Signaling, catalogue number 2507S) diluted in 1% Goat serum+ 0.1% Triton-X + TBS for 24 h at 4 °C. The sections were washed again with TBS + 0.1% Tween (3 × 5 min) and incubated in secondary antibody at 1:500 dilution (Alexa Fluor 488 conjugated to IgG goat anti-rabbit antibody, Invitrogen # A11008) diluted in 1% Goat serum+ 0.1% Triton-X + TBS for 1 h. Sections were washed in TBS + 0.01% Tween (3 × 5 min). Sections were then incubated in primary antibody (Synaptophysin,1:300, Cell Signaling, catalogue number D35E4) diluted in 1% Goat serum+ 0.1% Triton-X + TBS for 24 h at 4 °C. The sections were washed again with TBS + 0.1% Tween (3 × 5 min) and incubated in secondary antibody at 1:500 dilution (Alexa Fluor 594 conjugated to IgG goat anti-rabbit antibody, Abcam # Ab150080) diluted in 1% Goat serum+ 0.1% Triton-X + TBS for 1 h.

Sections were stained in 1:5000 DAPI diluted in 0.1 M PBS for 5 min, then washed in TBS for 5 min and mounted and coverslipped with PVA-DABCO for microscopy. Confocal images were obtained and analyzed using a 60X objective lens on an Olympus Fluoview confocal microscope. MetaMorph software (v.7) was used to quantify the % staining density of fluorescently labeled synaptophysin, PSD-95, and their colocalization, over a threshold intensity level for a region of the CA1 SR, as described in http://www.bioimagingsolutions.com/MolDev/metamorph_analysis.html.

### Data analysis and statistics

Evoked responses were analyzed offline using Clampfit 10 and Graphpad Prism 6 software. For PTP recordings, data were recorded 0–2 min post-tetanus, and for e-LTP, 15–20 min post-tetanus. Evoked EPSPs were averaged and expressed as a percentage of the average slope from pre-tetanus baseline recordings. For PPR analysis, EPSP amplitudes (mV) were expressed as a ratio of the second EPSP over the first EPSP. MiniAnalysis (version 6.0.9, Synaptosoft) was used to measure sEPSP events with minimal amplitude of 0.2 mV and minimal area of 3 mV*ms. Baseline was determined from a 1 ms average immediately prior to each event using ‘complex peak detection’ in MiniAnalysis. Data were expressed as ± SEM and assessed for significance using paired t tests, or one/two-way ANOVAs with Tukey or Student-Newman-Keuls post hoc analysis, where n denotes number of neurons in whole-cell patch clamp experiments or number of slices in LFP and VSD experiments. For all experiments, *n* = 8–10 neurons/slices for each group; power analysis was conducted on Statview to confirm sample size. Six to eight mice were used for each set of experiments.

## Results

### Impaired short-term synaptic plasticity in 3xTg-AD CA1 neurons

Based on known abnormalities in regulators of synaptic transmission and long-term plasticity in AD [21, 29, 45–48] we began by asking if analogous STP mechanisms are altered at early disease stages in the 3xTg-AD mouse model. Ideally, this is a period in which successful therapeutic intervention may still be achievable.

STP modulates synaptic strength and efficacy, and has roles in working memory, cognition, information processing and decision making [49–52]. There are several forms of STP, each with distinct signatures and functions. Post-tetanic potentiation (PTP), which lasts for seconds to minutes post-tetanus, reflects changes in Ca^2+^-dependent vesicle release probability, and is instrumental for stabilizing and modulating synaptic strength [53–55]. Early LTP (E-LTP) emerges ~ 20 min post-tetanus and involves post-translational modification of existing proteins such as glutamate receptors and second messengers [56–58]. Paired pulse facilitation (PPF) reflects Ca^2+^-dependent presynaptic STP. For comparison, late-LTP initiated 60 min post-tetanus requires gene transcription and de novo protein synthesis [59, 60].

**Fig. 1:**
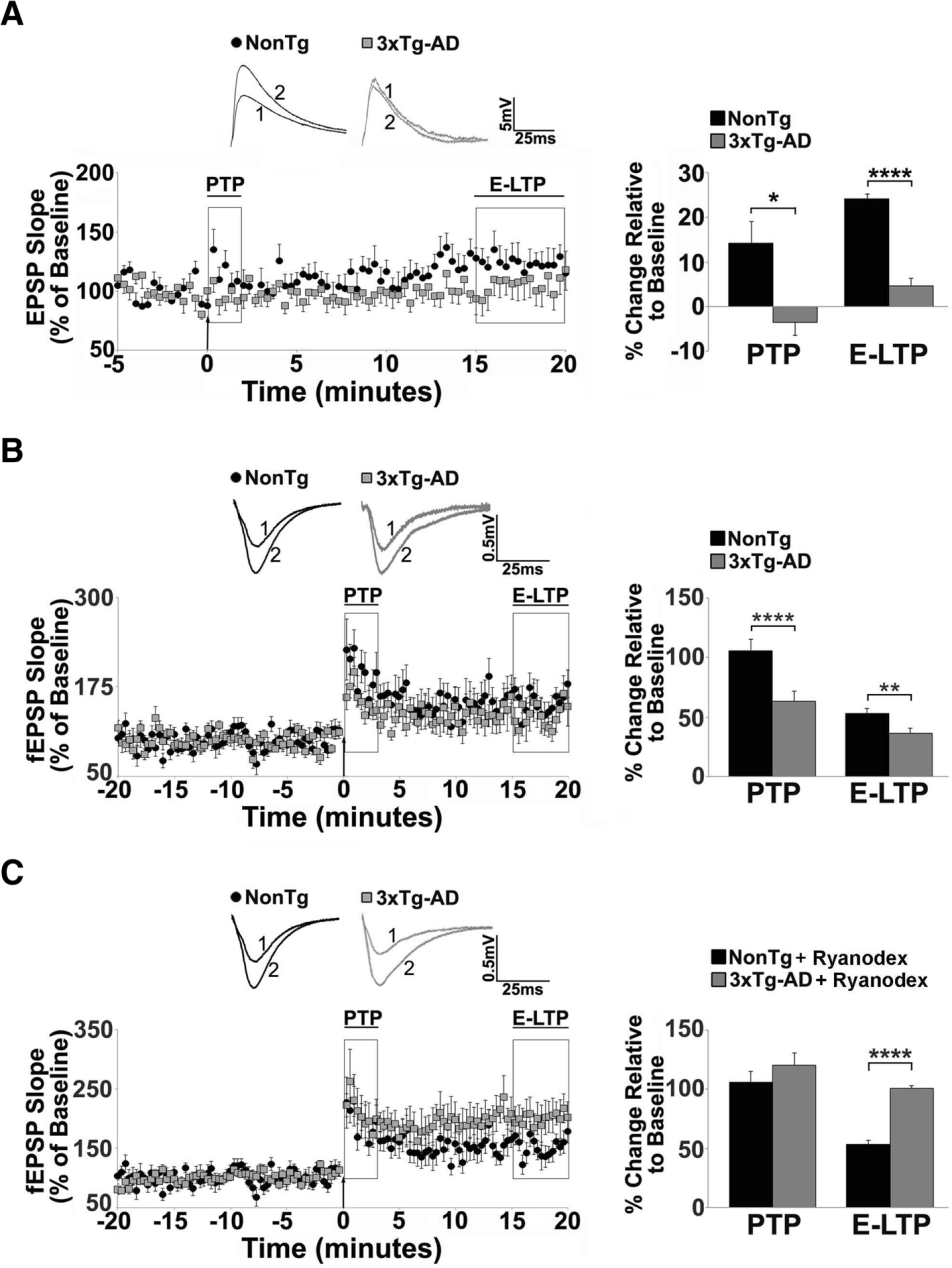
Short term plasticity deficits in 3xTg-AD CA1 region reflect RyR-Ca^2+^ abnormalities. **a-c** Left: Graphs show averaged time course of Schaffer collateral-evoked synaptic responses from NonTg (black circles) and 3xTg-AD (gray squares) in the hippocampal region using (**a**) whole cell patch clamp from CA1 pyramidal neurons (NonTg *n* = 10 neurons/6 mice; 3xTg-AD *n* = 10 neurons/6 mice) and (**b-c**) extracellular field potential recordings in CA1 stratum radiatum (NonTg *n* = 8 slices/5 mice; 3xTg-AD *n* = 8 slices/5 mice). Right: Bar graphs show averaged % change over baseline in the evoked response post-tetanus in 3xTg-AD (gray) compared to NonTg (black). Inset: Representative traces [1] before and [2] after tetanus from NonTg (black) and 3xTg-AD (gray) neurons. PTP and E-LTP are impaired in (**a**) individual neurons and (**b**) field potentials of 3xTg-AD CA1 neurons compared to NonTg neurons. **c** 30-day Ryanodex treatment restores or enhances PTP and E-LTP in 3xTg-AD CA1 circuits (*n* = 5 slices/5 mice; NonTg *n* = 5 slices/5 mice) . The arrow denotes the time of tetanus. Data are presented as Mean ± SEM; *p < 0.05, **p < 0.01 and ****p < 0.0001 represents significantly different from NonTg

As measured by whole cell patch clamp recordings from CA1 neurons in hippocampal slices, PTP and E-LTP from Schaffer collateral stimulation were severely impaired in 3xTg-AD mice vs NonTg mice (Fig. 1a). In NonTg neurons, PTP levels were 14.21 ± 4.9% over baseline (F_(3,40)_=4.12, *p* = 0.0123), while in 3xTg-AD neurons PTP was below baseline (− 3.48 ± 2.9%, Fig. 1a). Similarly, E-LTP was observed in NonTg CA1 neurons, but not in 3xTg-AD CA1 neurons (24.18 ± 1.2% vs. 4.72 ± 1.8% over baseline, respectively; F_(3,60)_=29.77, *p* < 0.0001). The implications for STP deficiency are substantial and impinge upon broader network signaling as well as the stability of long-term plasticity.

To investigate further at the local circuit level, extracellular field potential (fEPSP) recordings were used to measure STP in the CA1 stratum radiatum (SR) upon Schaffer collateral stimulation (Fig. 1b). While patch clamp recordings provide detailed information from single neurons, fEPSPs are sensitive to subthreshold signal integration and local network properties. This level of analysis reveals STP deficits in 3xTg-AD animals similar to those described through single neuron patch clamp recordings. It should be noted that the absolute magnitude of the responses between patch clamp and extracellular field potential recordings are on different scales since patch clamp responses are recorded in the soma of a single cell but generated in the dendrites and filtered by their cable properties, while field potential recordings are measured directly in the dendritic subfield and reflect ionic flux as measured in the extracellular space. In NonTg mice, PTP and E-LTP magnitudes were 105.99 ± 9.2% and 52.96 ± 3.9% over baseline, respectively, while PTP and E-LTP were blunted in 3xTg-AD mice (PTP: 63.18 ± 8.4% over baseline, F_(3,128)_ = 152.5, *p* < 0.0001; E-LTP: 36.48 ± 4.2% over baseline, F_(3,148)_ = 97.02, *p* < 0.0001).

Several mechanisms may underlie this deficit. Ca^2+^ dysregulation was first considered since it is fundamental to STP encoding and an integral component of AD pathogenesis. Prior studies have shown that abnormal ER Ca^2+^ release in AD models is prevented with dantrolene formulations which serve as negative allosteric modulators of RyR-evoked Ca^2+^ release [28, 45, 61]. We used this approach to determine if normalized Ca^2+^ signals in Ryanodex (a nanocrystalized formulation of dantrolene)-treated 3xTg-AD mice restores STP expression [61]. Here, PTP is enhanced (120.56 ± 10.2%) and E-LTP restored (100.94 ± 2.1% over baseline, Fig. 1c) in Ryanodex-treated 3xTg-AD mice relative to treated controls (PTP: *p* > 0.05; E-LTP: F_(3,148)_ = 421.9, *p* < 0.0001). Ryanodex in NonTg mice had no effect on STP expression (*p* > 0.05), consistent with its lack of effect on Ca^2+^ release in NonTg mice [61].

These findings reinforce the role of intracellular Ca^2+^ signaling in generating STP, and demonstrate that increased RyR-mediated Ca^2+^ release blunts STP in young 3xTg-AD mice prior to overt cognitive deficits and histopathology. To identify the signaling mechanisms underlying this deficit, we combined anatomical, physiological, Ca^2+^ imaging and network imaging approaches to characterize presynaptic, postsynaptic and circuit level functions in the hippocampus of control and AD mouse models. This approach was designed to identify early mechanisms of memory decline in neurological disorders [62–64].

### Abnormal presynaptic function in the 3xTg-AD hippocampus

#### Increased spontaneous vesicle release

**Fig. 2:**
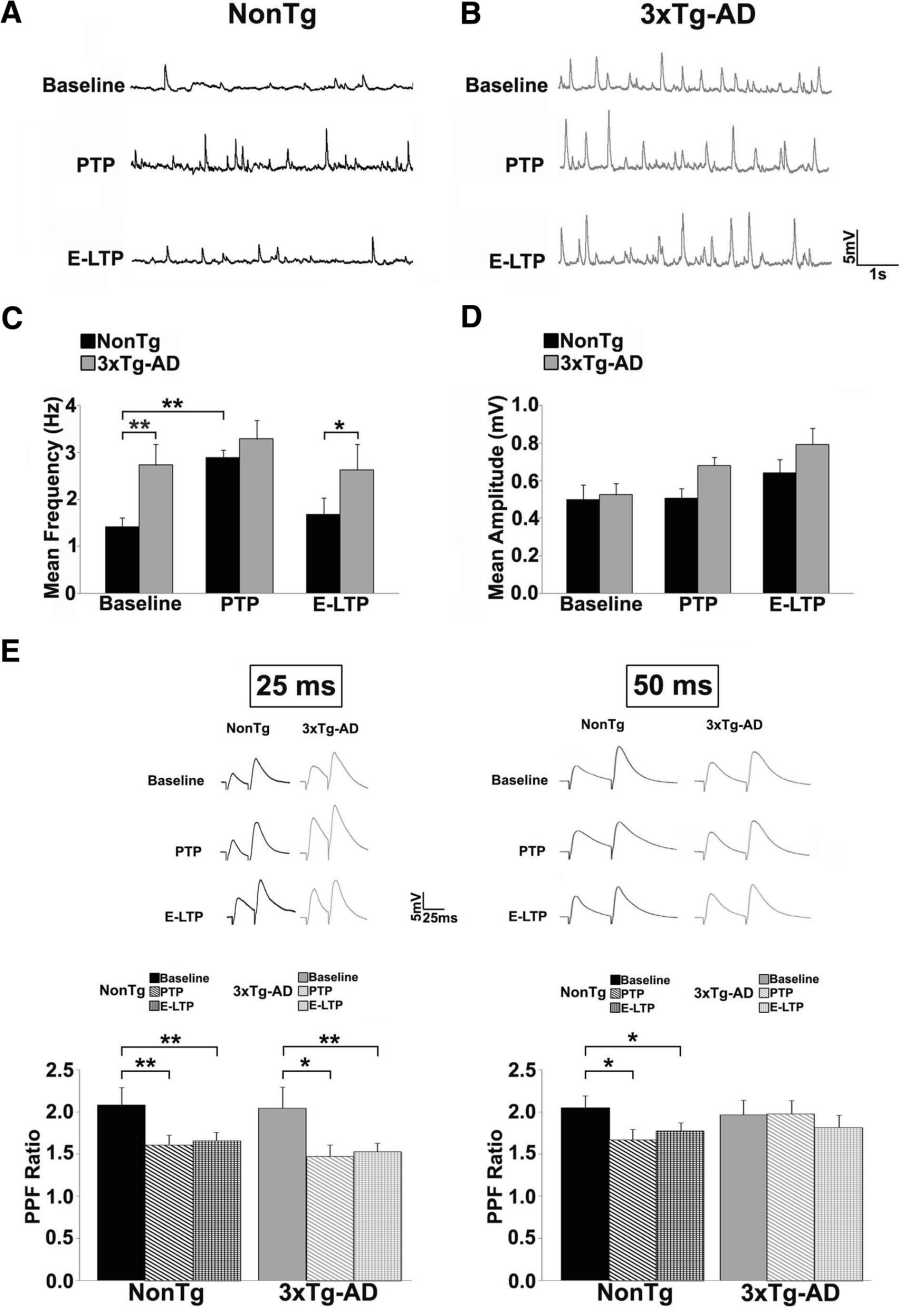
Impaired spontaneous and evoked presynaptic release properties in 3xTg-AD mice. Representative traces of sEPSPs from (**a**) NonTg (black) and (**b**) 3xTg-AD (gray) CA1 neurons at baseline, PTP, and E-LTP time-points. **c** Bar graphs show elevated averaged sEPSP frequency in 3xTg-AD (gray) neurons compared to NonTg (black) at baseline, and remains elevated during PTP and E-LTP. In NonTg neurons, sEPSP frequency is increased during PTP relative to its baseline, and returns to baseline during E-LTP. **d** sEPSP amplitude is not significantly different between NonTg and 3xTg-AD neurons at all time-points. Data are presented as Mean ± SEM; *p < 0.05 and **p < 0.01 represents significantly different from NonTg, **^^^*p* < 0.01 represents significantly different from pre-tetanus baseline. **e** Bar graphs show averaged paired pulse ratio (PPR) responses during baseline, PTP and E-LTP at 25 ms ISI (left) and 50 ms ISI (right) from NonTg (black, and patterned black) and 3xTg-AD neurons (gray and patterned gray). At the 50 ms ISI, the 3xTg-AD mice do not show the reduced PPR during PTP and E-LTP relative to baseline, as seen in the NonTg mice, suggesting that vesicle release probability is not further increased during these periods (NonTg *n* = 10 neurons/6 mice; 3xTg-AD *n* = 10 neurons/6 mice). Insets: Representative traces from NonTg (black) and 3xTg-AD (gray) at 25 ms and 50 ms. Data are presented as Mean ± SEM; *p < 0.05 and **p < 0.01 represent significantly different from pre-tetanus baseline

Concomitant with PTP expression are changes in presynaptic spontaneous neurotransmitter release that manifests as an increase in the frequency of vesicle release in this form of plasticity [65, 66]. Based on the STP defects in AD mice, we asked if the LTP-inducing tetanus differentially alters spontaneous excitatory postsynaptic potentials (sEPSP) properties in 3xTg-AD mice during PTP and E-LTP epochs. Again, there is a clear distinction in presynaptic release patterns between control and AD neurons (Fig. 2a-d, F_(3,23)_=7.09, *p* = 0.0015). During PTP, sEPSP frequency is increased in NonTg CA1 neurons (Fig. 2a,c; paired t_(5)_=5.05, *p* = 0.003), consistent with the enhanced vesicle release probability that occurs during this plasticity phase. However, in 3xTg-AD neurons, there is no further enhancement in release frequency during PTP over baseline (*p* > 0.05), and sEPSP frequency is now similar between NonTg and 3xTg-AD neurons. During E-LTP, NonTg sEPSP frequency returned to pre-tetanus baseline, yet frequency remained elevated, similar to their baseline, in 3xTg-AD neurons (2B,C). Due to higher baseline spontaneous release in 3xTg-AD mice, the lack of further post-tetanus increases can reflect a ‘ceiling’ effect on vesicle release due to enhanced Ca^2+^ leak in terminals and/or a reduction in vesicle pools. sEPSP amplitude did not differ between NonTg and 3xTg-AD neurons (Fig. 2d) during PTP or E-LTP, suggesting that postsynaptic mechanisms are not a prevailing mechanism here.

#### Abnormalities in synaptically-evoked neurotransmitter release

The increased spontaneous release and blunted potentiation during PTP at 3xTg-AD CA1 synapses indicate dominant presynaptic defects. To pursue this hypothesis, we investigated evoked presynaptic plasticity mechanisms and asked if PPF is altered at the CA3-CA1 synapse during PTP or E-LTP (Fig. 2e). Baseline (pre-tetanus) PPF (measured as the ratio of the 2nd response over the 1st) was similar between NonTg and 3xTg-AD mice at all interstimulus intervals (ISI; 25 ms, 50 ms, and 200 ms). At the 25 ms ISI, NonTg and 3xTg-AD animals showed similarly decreased PPF ratios relative to their own baselines during PTP (left; paired t_(29)_=2.18, *p* = 0.037; t_(29)_=2.77,*p* = 0.009; respectively); and E-LTP (t_(59)_= 3.13,*p* = 0.002, paired t_(38)_=3.11, *p* = 0.01, respectively), as would be expected under normal conditions of increased release probability and residual presynaptic Ca^2+^. Since increased release probability is positively associated with PTP expression [66], this manifests as a decrease in the paired pulse ratio [55, 67–69]. Consistent with this, in NonTg neurons, PPF ratios at the 50 ms interval were decreased during PTP and E-LTP (t_(29)_=2.54, *p* = 0.016; t_(59)_= 2.54, *p* = 0.01 respectively). However, in the 3xTg-AD neurons, PPF ratios at 50 ms ISI did not change during PTP or E-LTP relative to baseline, indicating unchanged vesicle release probability. At 200 ms ISI there were no changes in PPF ratios in either NonTg or 3xTg-AD CA1 neurons during PTP or E-LTP (data not shown), reflecting restoration of resting Ca^2+^ levels. Thus, for spontaneous and evoked activity, 3xTg-AD mice demonstrate abnormal Ca^2+^-dependent presynaptic signaling and STP early in the disease process.

#### Reduced presynaptic vesicle stores within active zones can account for synaptic plasticity deficits in AD

Deficits in presynaptic plasticity can be attributed to a reduction in available neurotransmitter vesicles within active zones of CA3 terminals. Physiological events that align with this are enhanced presynaptic Ca^2+^ tone and associated excessive spontaneous vesicle release; conditions which are met in the AD mice [28, 29]. To verify, electron microscopy (EM) studies were conducted to quantify dense core vesicles in CA3 terminals within the docked zone (within 50 nm from the presynaptic membrane), reserve zone (between 50 and 300 nm), and resting pool (beyond 300 nm), and postsynaptic density length (PSD) [41].

**Fig. 3:**
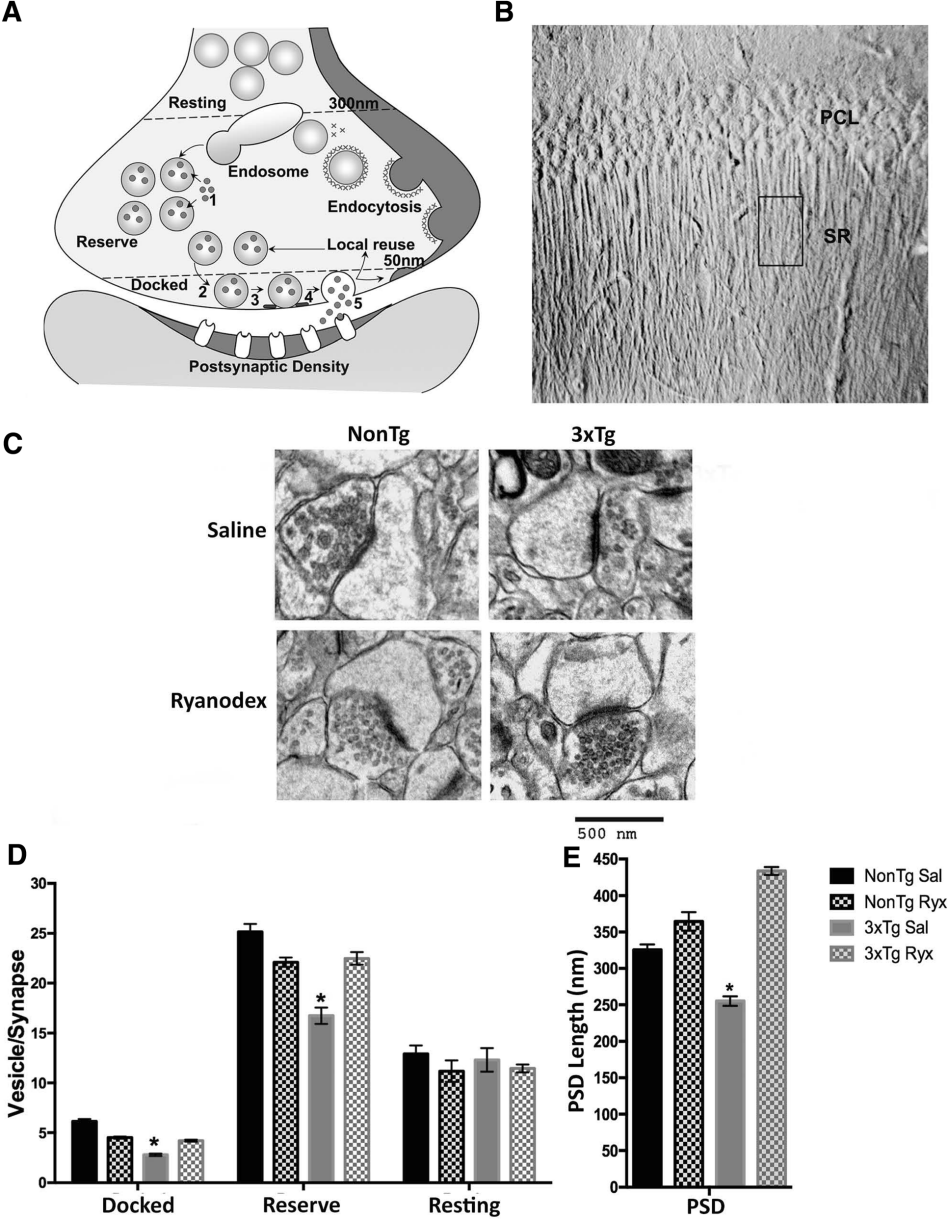
3xTg-AD mice have fewer synaptic vesicles in active zones and decreased PSD length. **a** Diagram showing synaptic vesicle cycle including [1] neurotransmitter uptake, [2] docking, [3] priming, [4] fusion and [5] release, and vesicle classification based on distance from the presynaptic membrane (docked: 0-50 nm, reserve: 50-300 nm, resting: > 300 nm) [41]. **b** DIC image showing region of interest within the CA1 stratum radiatum (SR) from which ultrathin sections were obtained for EM; PCL: Pyramidal Cell Layer. **c** Representative electron micrographs from saline- and Ryanodex-treated NonTg and 3xTg-AD asymmetric synapses (saline: NonTg *n* = 40 micrographs/5 mice; 3xTg-AD *n* = 56 micrographs/7 mice. Ryanodex treated: NonTg *n* = 36 micrographs/4 mice; 3xTg-AD *n* = 45 micrographs/5 mice). Scale bar: 500 nm, direct magnification: 30,000X, PSD: Postsynaptic Density. Bar graphs comparing (**d**) number of synaptic vesicles per synapse and (**e**) PSD length observed in NonTg and 3xTg-AD mice. Data are presented as Mean ± SEM; *p < 0.01 represents significantly different from NonTg Sal group

An important finding is that within the docked and reserve zones, 3xTg-AD mice had significantly fewer vesicles per terminal than NonTg mice (F_(3,1546)_ = 63.2, *p* < 0.001; F_(3,1191)_ = 62.0; *p* < 0.001; respectively), yet there were no differences among groups in the resting zone (F_(3,1018)_ = 1.2; *p* = 0.3), suggesting that the depleted stores are restricted to the regions with a higher turnover that are actively engaged in release and recycling (Fig. 3c-d). Thus, in addition to increased vesicle release associated with increased Ca^2+^ tone, there is a likely deficit in replenishing stores in active zones, a function regulated by RyR-Ca^2+^ [70–73]. This novel finding of reduced vesicle content in docked and reserve pools is sufficient to account for the synaptic plasticity and signaling deficits in AD mice, and may be one of the earliest features of synaptic pathophysiology described to date. Due to the known Ca^2+^-dependence on synaptic vesicle release and replenishment, we next asked if normalizing RyR-Ca^2+^ signaling with 30-day Ryanodex treatment (10 mg/kg; i.p., [61]) would restore or increase the population of docked and reserve pools. As shown in Fig. 3 C/D, this treatment increased vesicle numbers in the docked and reserve pools from the 3xTg-AD/Ryx group to the same levels as the NonTg/Ryx group (Scheffe post hoc analysis; *p* = 0.47 and *p* = 0.99, respectively). Furthermore, 3xTg-AD mice show shorter PSD lengths compared to NonTg mice (t_(587)_ = 6.2, *p* < 0.001), possibly reflecting a compensatory response to reduce excess postsynaptic stimulation related to increased sEPSP frequency (Fig. 3c, e). This pattern is also reversed in the 3xTg-AD/Ryx treatment group, such that the PSD length is now greater than in the remaining groups (*p* < 0.01for all comparisons). While the functional significance of this is under investigation, the discovery that the ultrastructure of the synapse and levels of presynaptic vesicle are altered in early stages of AD, and show considerable reversal of deficits upon normalizing RyR-Ca^2+^ signaling, demonstrates a highly plastic and potentially salvageable synaptic phenotype that may directly act to prevent the degenerative memory loss in AD.

### Increased synaptically-evoked Ca^2+^ responses in 3xTg-AD CA1 dendrites

Despite the fundamental role Ca^2+^ signaling plays in synaptic plasticity and memory encoding [74–76], Ca^2+^ responses generated during the tetanus or during STP have yet to be studied in cognitive disease models such as AD. To address this, we used 2-photon Ca^2+^ imaging of fura-2 filled CA1 neurons to compare postsynaptic Ca^2+^ signals generated by an LTP-inducing tetanus (2x100Hz, 10s apart) to determine if evoked Ca^2+^-signals are altered at critical plasticity-encoding epochs during AD pathogenesis.

**Fig. 4:**
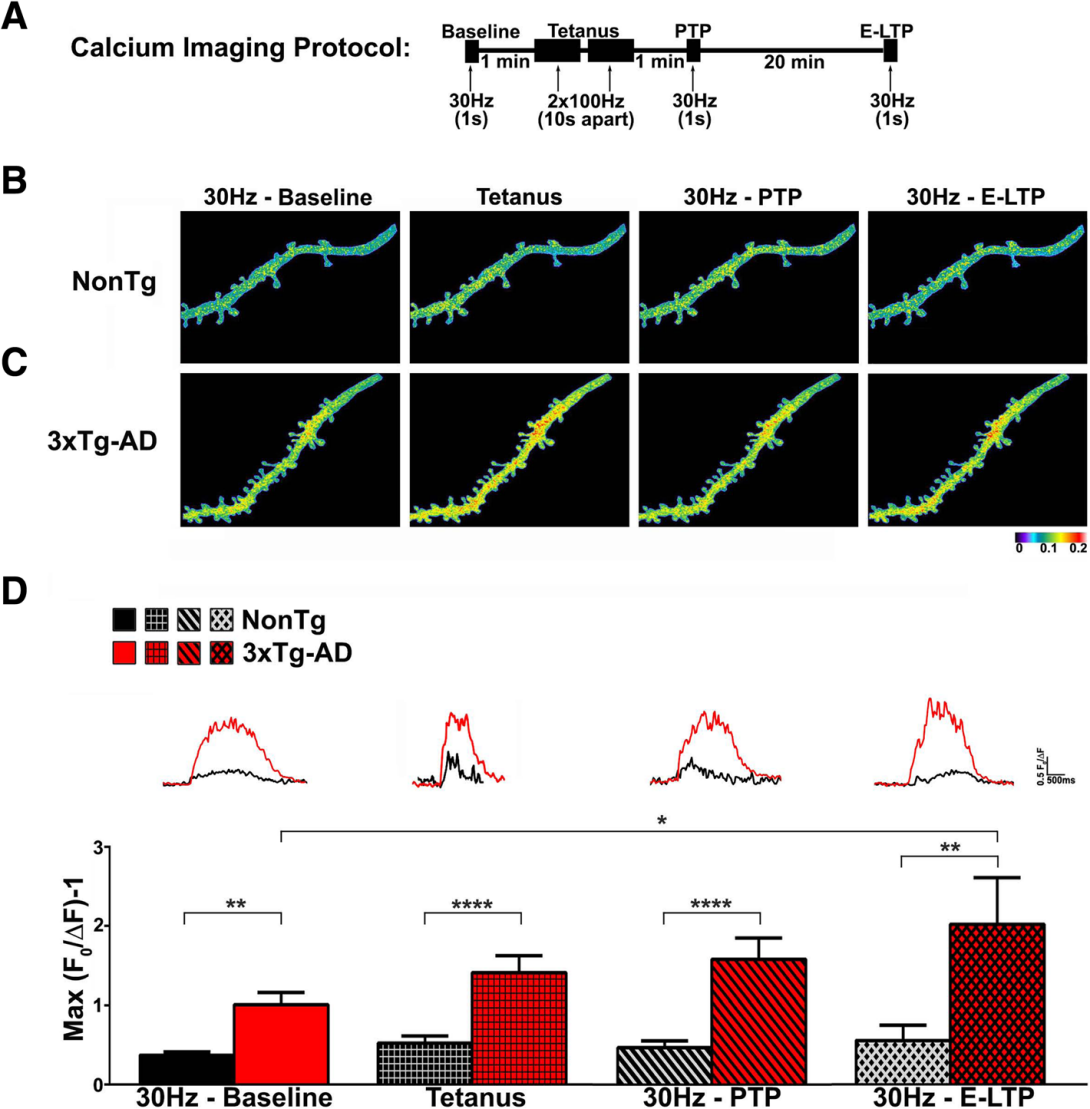
Continuously increasing synaptically-evoked Ca^2+^ responses in 3xTg-AD CA1 neurons during and after tetanus. **a** Schematic showing the synaptic stimulation and Ca^2+^ imaging protocol. **b-c** Representative pseudocolored Ca^2+^ images of dendritic segments from (**b**) NonTg and (**c**) 3xTg-AD neurons at baseline, tetanus, PTP and E-LTP showing increased Ca^2+^ responses in 3xTg-AD neurons. Colors correspond to relative Ca^2+^ changes indicated by the bar below. (**d**) Bar graphs show increased Ca^2+^ responses in 3xTg-AD dendrites compared to NonTg at baseline, tetanus, PTP and E-LTP (NonTg *n* = 10 neurons/6 mice; 3xTg-AD *n* = 10 neurons/6 mice). Insets: Representative traces of Ca^2+^ responses from NonTg (black) and 3xTg-AD (red) dendrites. Data are presented as Mean ± SEM; **p < 0.01 and ****p < 0.0001 represent significantly different from NonTg; *p < 0.05 represents significantly different from pre-tetanus baseline

Baseline postsynaptic Ca^2+^ responses in CA1 stratum radiatum were evoked by Schaffer collateral stimulation (30 Hz, 1 s train). This stimulus generates stable physiological Ca^2+^ responses without altering vesicle release properties [21]. Evoked Ca^2+^ responses were measured at four time-points – pre-tetanus (baseline), during tetanus (2x100Hz, 10s apart), 1 min post-tetanus (PTP) and 20 min post-tetanus (E-LTP) (Fig. 4a). L-LTP (50–60 min after tetanus) responses were not measured due to potential washout of intracellular messengers. At each time-point, evoked Ca^2+^ responses were 3–4-fold greater in dendrites and spines of 3xTg-AD neurons than in NonTg neurons (Fig. 4b-d, baseline: F_(3, 52)_=25.91, tetanus: F_(3,52)_=19.83, PTP: F_(3,52)_=16.55, E-LTP: F_(3,52)_=5.57, *p* < 0.001). In NonTg neurons, post-tetanus Ca^2+^ responses did not differ from baseline regardless of time-point; however in 3xTg-AD neurons, Ca^2+^ responses steadily increased at each test point, with significance reached at E-LTP vs. baseline (F_(1,64)_=39.34, *p* < 0.0001). This augmentation suggests a Ca^2+^ signaling metaplasticity in the AD brain that is not normally present.

The Ca^2+^ and plasticity abnormalities in 3xTg-AD neurons appear activity-dependent, as there were no differences in passive membrane properties (resting membrane potential or input resistances), at baseline or post-tetanus (*p* > 0.05 for either strain). Voltage responses to injected current steps were also not different between NonTg and 3xTg-AD neurons pre- or post-tetanus (*p* > 0.05 for either strain). Data not shown.

### Excessive dendritic Ca^2+^ responses mediate loss of mushroom spines and synaptic contacts in AD hippocampal neurons

Stable presynaptic-postsynaptic connections and mature dendritic spine architecture are necessary structural elements for proper plasticity encoding and expression. The structural stability of the presynaptic – postsynaptic microdomain is maintained through a variety of scaffold pathways largely involving adhesion molecules to stabilize synapses [77–79]. For example, Ca^2+^-regulated cell adhesion molecules, such as N-cadherin, maintain the close junctions between pre and postsynaptic elements necessary for efficient synaptic communication and plasticity [80–82]. Stable spine formation associated with long term memory changes and synaptic efficacy is supported by actin cytoskeleton reconfiguration [83, 84]. Ca^2+^ dynamics within synaptic compartments play a key role in guiding structural fate [85–87] and are implicated in the loss of stable mushroom spines in neurons from AD mouse models [33, 88, 89]. Dendritic spines are not homogenous and are grouped as mushroom, thin, or stubby, each with distinct morphology and function. While thin spines are considered highly plastic, stubby spines are critical during postnatal development, and mushroom spines are relatively stable and incorporated into established neuronal networks and serve memory functions [37, 43, 79, 90].

**Fig. 5:**
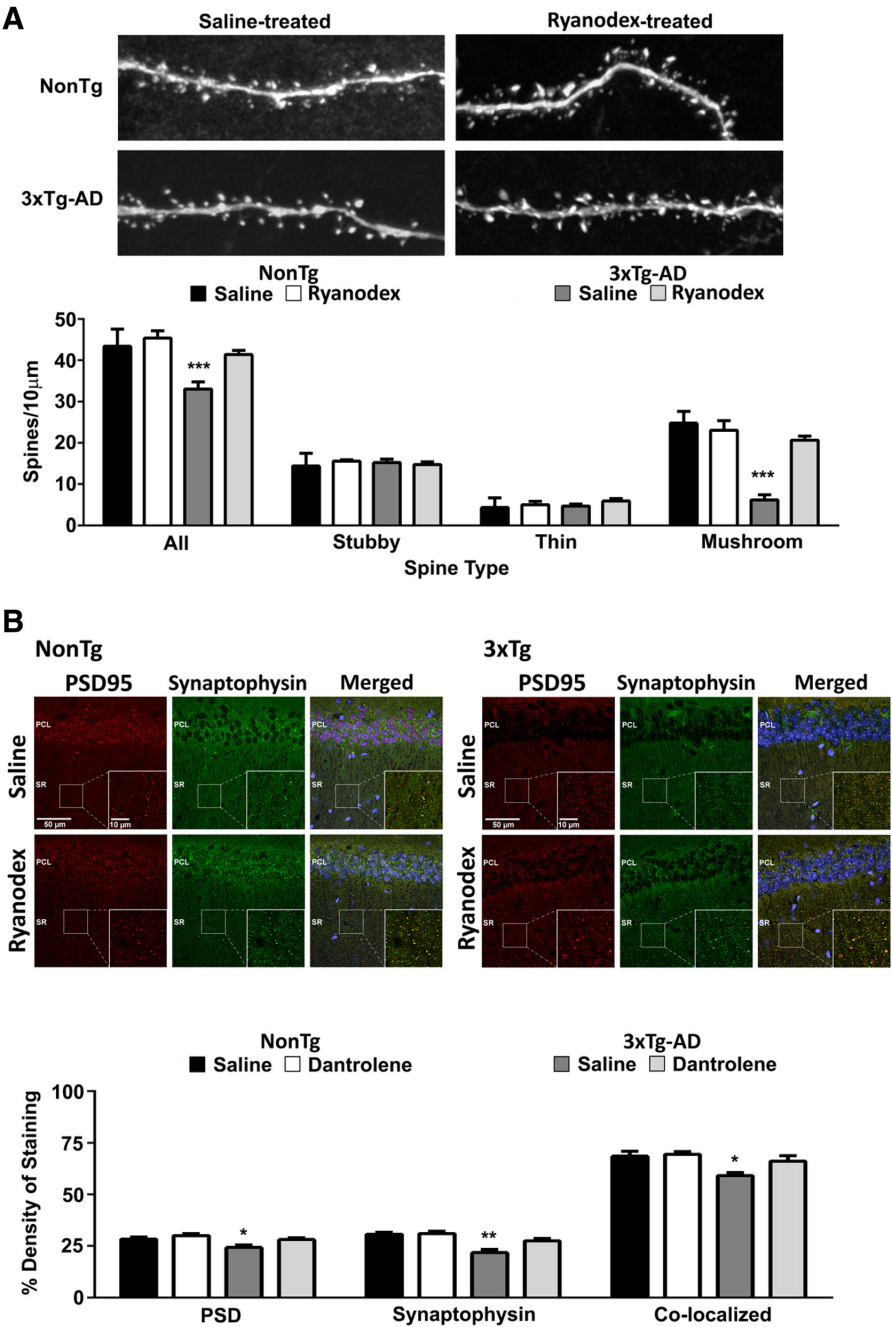
Loss of mushroom spines and synaptic integrity is reversed by normalized RyR-Ca^2+^ in 3xTg-AD neurons. **a** Top: Images show Lucifer Yellow-filled dendrites and spines from saline- and Ryanodex-treated NonTg and 3xTg-AD CA1 neurons. Bottom: Bar graphs show a significant loss of mushroom spines in the 3xTg-AD mice, and a restoration of mushroom spine number after Ryanodex treatment in 3xTg-AD neurons. (Saline treated: NonTg *n* = 8 neurons/3 mice; 3xTg-AD *n* = 6 neurons/3 mice. Ryanodex treated: NonTg *n* = 4 neurons/3 mice; 3xTg-AD *n* = 8 neurons/3 mice; 2–5 dendritic primary or secondary branches from each cell). **b** Top: Confocal images (40x, single plane, axial resolution < 0.3 μm) show colocalized immunolabeling of postsynaptic PSD (postsynaptic density, red) and presynaptic (synaptophysin, green) proteins at the CA3-CA1 synapses from saline- and Ryanodex-treated NonTg and 3xTg-AD mice. Inset: higher magnification (100x) detailing synaptic labeling patterns in each condition, with yellow fluorescence indicating close localization of pre- and postsynaptic markers in the merged images. Bottom: Bar graphs show a reduction of synaptophysin and PSD95 labelling, and colocalization of pre- and postsynaptic proteins in the saline-treated 3xTg-AD mice, and that Ryanodex-treatment results in the recovery of these synaptic proteins in 3xTg-AD mice, and has no effects in the NonTg mice. (NonTg *n* = 36 slices/6 mice for each treatment condition; 3xTg-AD *n* = 36 slices/6 mice for each treatment condition). Data are presented as Mean ± SEM; *p < 0.05, **p < 0.01 and ***p < 0.001 represent significantly different from saline-treated 3xTg-AD

Based on the steep increase in synaptically-evoked Ca^2+^ within spines of 3xTg-AD mice, we asked if this affects the density and proportion of the varying spines types in CA1 neurons. We filled CA1 neurons from fixed hippocampal slices with Lucifer Yellow dye, generated 3D images using confocal microscopy and deconvolution software, and compared spine number and morphological type between the NonTg and AD mice [44, 91–93]. As previously described in older AD mice [94], we found a significant reduction in the total number of spines in younger 3xTg-AD mice (F_(3,100)_=10.14; *p* < 0.001), and a selective reduction in mushroom spines (F_(3,100)_=8.3; *p* < 0.001); the density of thin and stubby spines were not different (*p* > 0.05) (Fig. 5a). We next tested the role of RyR-Ca^2+^ stores as an underlying mechanism driving the loss of the stable mushroom spines in mice treated with Ryanodex (as above). Consistent with previously described RyR-mediated synaptic deficits in AD, the 3xTg-AD mice treated with the negative allosteric RyR modulator had normal spine density and restored mushroom spine density (Fig. 5a). Notably, this treatment had no effect on the NonTg mice, suggesting this is not related to a general effect on mushroom spines, and speaks to normalization of an aberrant Ca^2+^ source. This is consistent with the mechanism of action of Ryanodex, which restores normal function to leaky RyR, but has little effect on properly functioning channels [61].

The structural connectivity of synapses, as measured by the colocalization of fluorescently-labeled presynaptic and postsynaptic proteins (synaptophysin and PSD95, respectively) was examined in CA1 using immunohistochemical labeling and confocal microscopy (Fig. 5b). The density of each fluorophore was measured individually, and the degree of colocalization was measured to ascertain pre and postsynaptic components of synaptic pathology, and determine if there is a deficit in synaptic adhesion independent of synaptophysin and PSD95 levels. These analyses were conducted in saline-treated and Ryanodex-treated NonTg and 3xTg-AD mice to determine if deficits could be reversed by normalizing RyR-Ca^2+^ signaling. There was a significant decrease in synaptophysin density in the saline-treated 3xTg-AD mice only (F_(3,72)_=5.7; *p* < 0.01); Ryanodex-treated 3xTg-AD mice demonstrated normal synaptophysin levels compared to NonTg controls (*p* > 0.05) where Ryanodex had no effect. The pattern was very similar for PSD95 staining, with a significant decrease in saline-treated 3xTg-AD mice (F_(3,114)_=2.37; *p* < 0.05) that was normalized with Ryanodex treatment. Perhaps most relevant, the density of colocalized synaptic proteins was significantly reduced in the saline-treated 3xTg-AD mice relative to NonTg (F_(3,90)_=2.5; *p* < 0.05); Fig. 5b). As above, we questioned the role of Ca^2+^ dysregulation as a contributing factor in this deficit. In the Ryanodex-treated mice, we see a restoration of synaptic integrity, as well as normalized PSD95 and synaptophysin immunostaining levels in the 3xTg-AD mice, with no effects in NonTg mice.

### Defect in CA1 network plasticity identified in stratum oriens

**Fig. 6:**
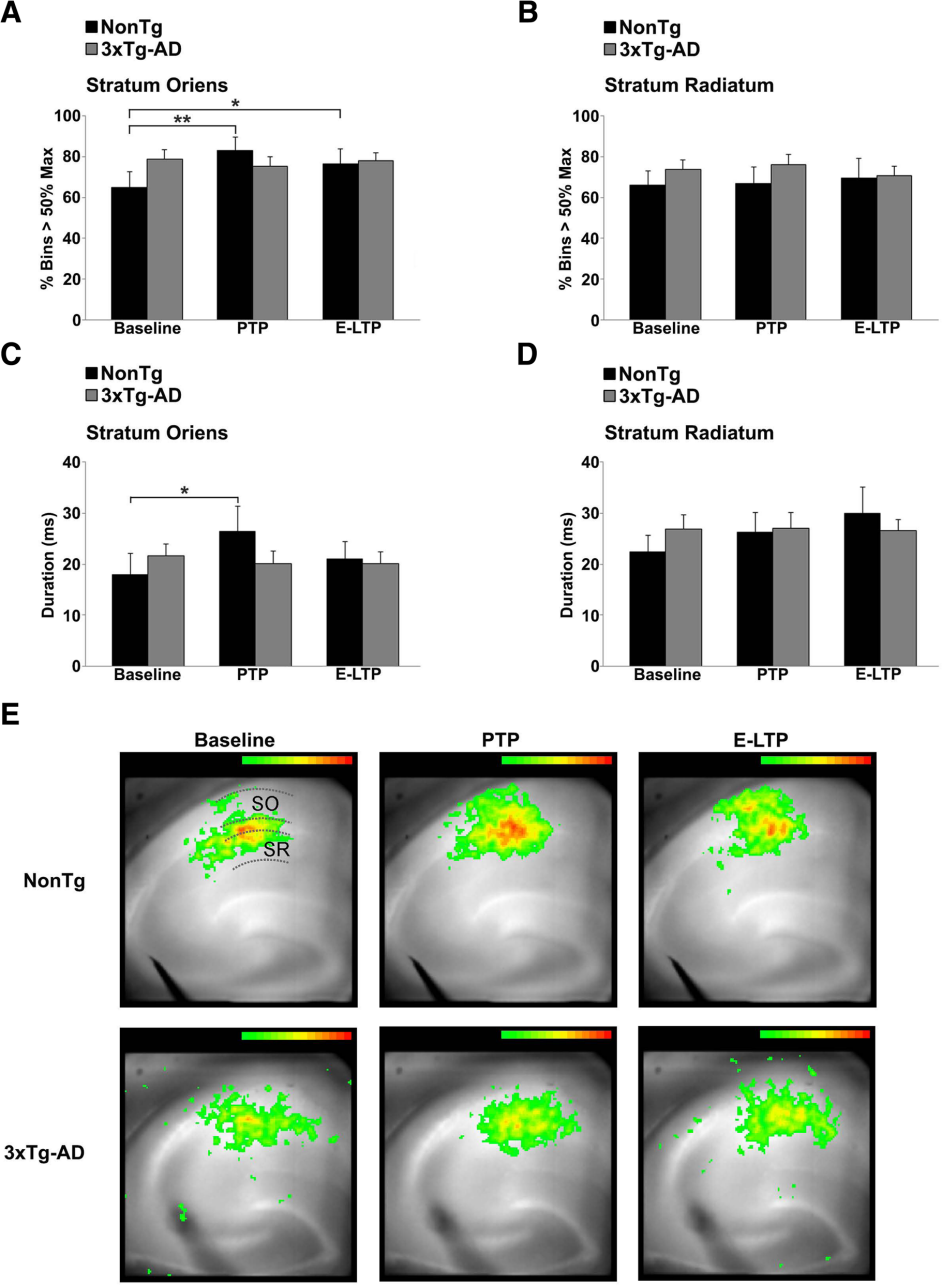
Network-level STP deficits in the CA1 SO subfield of 3xTg-AD hippocampus. Bar graphs show spread (**a-b**) and duration (**c-d**) of strong depolarizing optical signals across the CA1 subfield in response to a single pulse to CA3 at baseline, PTP and E-LTP in the SO (**a**, **c**) and SR (**b**, **d**) from NonTg (*n* = 9 mice, 9 slices) and 3xTg-AD mice (*n* = 11 mice, 11 slices). There are no changes in spread or duration of optical signals in 3xTg-AD hippocampus after tetanus, compared to increased spread and duration of optical signals in NonTg hippocampus. **e** Pseudocolored VSD images showing the spread of optical signals in NonTg and 3xTg-AD hippocampus during baseline, PTP and E-LTP. The SO, SP, and SR regions are indicated by dashed lines in the NonTg/Baseline image. Optical signals > 50% over baseline are shown. Data presented as Mean ± SEM; *p < 0.05 and **p < 0.01 represent significantly different from pre-tetanus baseline

The above-described deficits reveal early and profound alterations in cellular and local circuit functions in AD models; however, to make more relevant links to cognitive decline, a broader examination of hippocampal network function is needed. Thus, wide-field VSD imaging approaches were used to determine if the cellular defects in 3xTg-AD mice translate to abnormal signal propagation across subfields of the Schaffer collateral - CA1 network [95–97]. Although neural activity and glial responses contribute to VSD signals [98], VSD imaging is a powerful technique to monitor circuit activity across brain networks. In AD mouse models, VSD imaging has previously been used to examine signaling in the dentate gyrus [99], but to our knowledge, this is the first VSD study to examine AD-related CA1 network deficits in concert with electrophysiological and Ca^2+^ responses. Hippocampal slices were loaded with the VSD RH155 and spatial and temporal properties of depolarizing responses evoked by a single pulse to CA3 afferents were measured at baseline, PTP and E-LTP time points over the CA1 stratum radiatum (SR) and stratum oriens (SO) subfields (Fig. 6). There was no difference in the spatial properties of depolarization within the SR or SO between NonTg and 3xTg-AD mice at baseline, nor was there a difference post tetanus in the CA1 SR between NonTg and 3xTg-AD mice (Fig. 6b). However, when comparing pre- vs post-tetanus responses in the SO, only the NonTg mice demonstrated an increased spatial area of depolarization (F_(2,12)_=5.06, *p* = 0.015) at both the PTP and E-LTP time points (*p* < 0.01 and *p* < 0.05 respectively, Fig. 6a). In contrast, the lack of change in the SO depolarization area in 3xTg-AD mice at these time points suggests blunted postsynaptic plasticity expression (Fig. 6a).

Given the Hebbian nature of hippocampal plasticity encoding, we also asked how the tetanus affects temporal properties of depolarizing synaptic responses in the CA1 subfield of NonTg and 3xTg-AD mice. Again, no significant changes were observed in SR during baseline, PTP or E-LTP in NonTg and 3xTg-AD mice (Fig. 6d). However, there was a significant increase in the duration of depolarizing responses in NonTg CA1 SO during PTP (F_(2,12)_=3.72, *p* = 0.039; Fig. 6c). There were no corresponding temporal increases in the 3xTg-AD SO (Fig. 6c). Thus, in the NonTg hippocampus we observed, post-tetanus, increased spatial and temporal properties of the CA1 response serving to promote Hebbian plasticity, synaptic potentiation and temporal summation in the CA1 SO region in NonTg mice. Since our electrophysiology and Ca^2+^ imaging experiments focused on SR, and not SO, these results were unexpected and present new opportunities to investigate sub-field specific deficits in plasticity encoding in AD. Intriguingly, the SO may be a locus of network-level plasticity deficits in the earliest stages of AD.

## Discussion

**Fig. 7:**
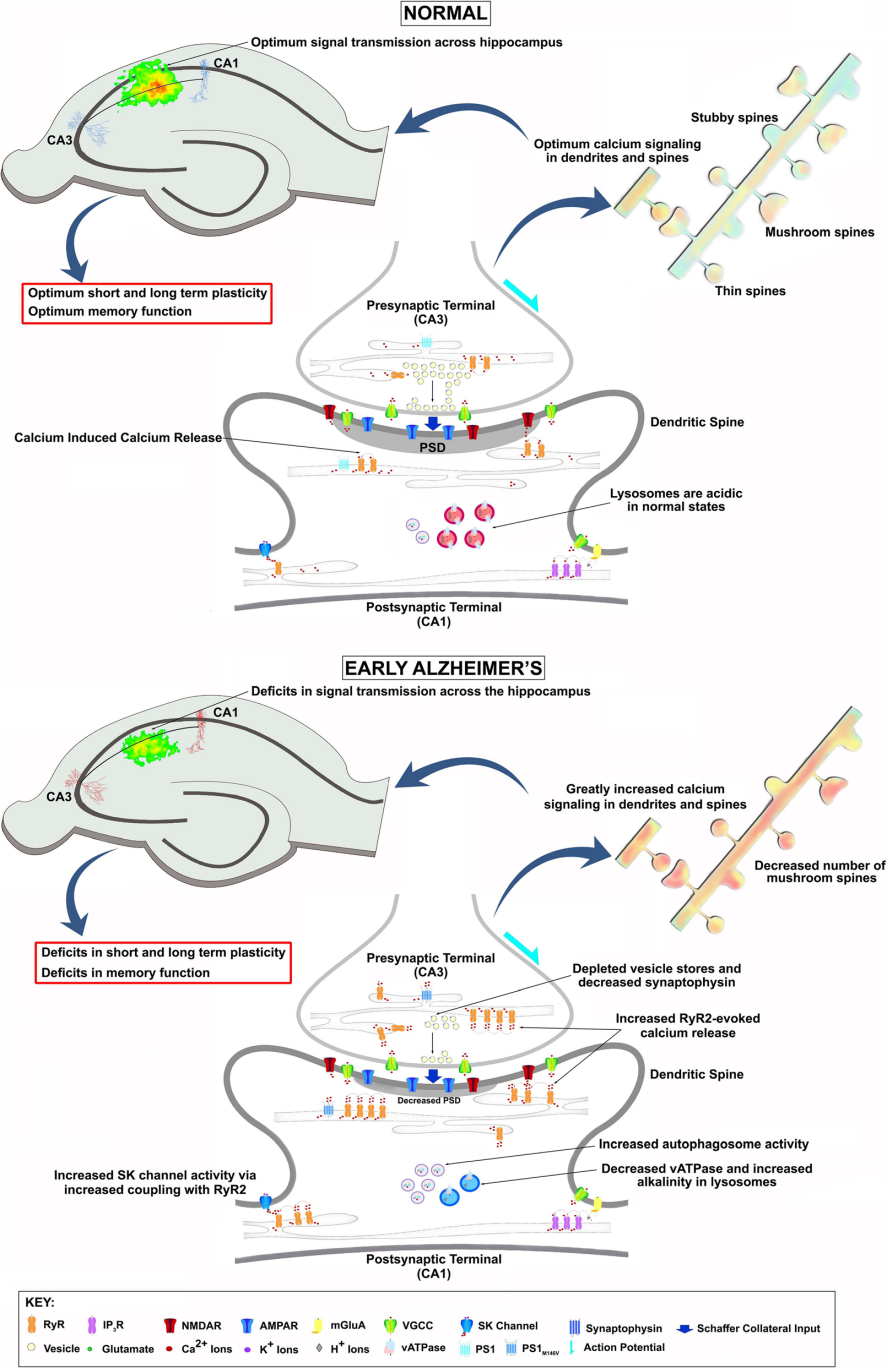
Proposed role of RyR-Ca^2+^ signaling in early impairment of short term plasticity at the CA3-CA1 synapse in Alzheimer’s disease. Normal: In presynaptic CA3 terminals, RyR-evoked Ca^2+^-Induced-Ca^2+^-Release (CICR) can trigger spontaneous neurotransmitter release. During high frequency activity (such as a train of action potentials), CICR is evoked by voltage-gated Ca^2+^ influx to increase residual Ca^2+^ levels and release probability. Postsynaptically, in CA1 terminals, NMDAR-mediated Ca^2+^ signals are amplified by RyR-CICR in dendritic spines, a phenomenon required for plasticity induction. In other plasticity pathways, the mGluA-PLC pathway generates IP_3_, activating IP_3_R. Activation of IP_3_Rs produces regenerative Ca^2+^ waves that support plasticity and gene expression. RyR-Ca^2+^ also activates SK channels that modulate membrane excitability and frequency of action potential firing. Thus, optimum levels of Ca^2+^ signaling and synaptic plasticity proteins like synaptophysin and PSD in dendritic spines support maintenance of spine structure and signal transmission across the hippocampus, generating optimal levels of plasticity and normal memory function. Early AD: Presynaptically, increased RyR expression greatly increases CICR. Increased CICR alters vesicle cycling and cause depletion of vesicles from the readily releasable pool as well as the reserve pool. While this can be initially remedied by increasing neurotransmitter synthesis and vesicle cycling, such maladaptive mechanisms can potentially cause metabolic and oxidative stress leading to synapse loss. Postsynaptically, increased RyR-CICR can decrease PSD lengths and weaken synaptic integrity resulting in the loss of dendritic spines, specifically mushroom spines required for synaptic plasticity. Increased RyR-CICR can also increase SK channel activity which decreases neuronal excitability and increases threshold for induction of synaptic plasticity. Thus, greatly increased Ca^2+^ signaling and loss of proteins supporting synaptic plasticity result in the loss of synaptic integrity and dendritic mushroom spines and decreased signal transmission across the hippocampus. These results in early deficits in short and long term synaptic plasticity that ultimately causes memory impairments

While preserving cognitive function is the ‘holy grail’ for AD therapeutics, the proximal mechanisms driving memory loss have remained elusive. Sustainable long-term memory encoding relies upon Ca^2+^-dependent short-term plasticity to establish synapse specificity and stably transform short-term into long-term memory through synaptic tagging and capture [7, 100]. RyR-evoked Ca^2+^ signaling plays a specific key role in the timing and strength of synaptic tagging; RyR activation *during* the tetanus increases the synaptic tag duration and durability, thus enabling LTP associativity; in contrast, if RyR channels are active *prior to* plasticity induction, as demonstrated in the AD models, the associative learning window is shortened and plasticity resilience is reduced [8, 101]. Thus, one possibility suggested by the present study is that increased synaptic RyR activity weakens the critical tagging process needed for transforming and stabilizing long-term memory formation, resulting in impaired memory performance such as that observed in these 3xTg-AD mice at 3 months of age [9, 11, 12]. Notably, the ER-Ca^2+^ signaling abnormalities and memory deficits precede detectable amyloid and tau pathology in AD [17, 31, 45, 102]. Thus, we feel we have identified a critical plasticity signaling deficit during AD pathogenesis that has significant implications for memory formation (Fig. 7).

The increased synaptically-evoked Ca^2+^ responses described in young AD mice are especially pronounced within dendrites and spines, at a time and location where NMDA receptor activation triggers further RyR-mediated Ca^2+^ release from ER stores [21, 29, 103]. These altered postsynaptic Ca^2+^ responses can initiate multiple pathogenic cascades, resulting in a range of defects from ultrastructural abnormalities to network propagation deficits, as detailed in this study. A critical new finding revealed here is the reduction in neurotransmitter vesicle stores within presynaptic active zones in the AD models, which is likely is associated with the increased spontaneous vesicle release resulting from enhanced RyR-Ca^2+^ tone in terminals, and thus contributing to vesicle depletion. Under these compromised conditions, vesicle reserves are rapidly diminished during activity-demanding events such as a tetanus [28, 29] and contribute to weakened synaptic plasticity. While increased frequency of sEPSPs, synaptic depression mediated by excess RyR-Ca^2+^, and presynaptic protein pathology in AD mouse models have previously been described [28, 45, 61], until now, functional presynaptic deficits were largely inferred. Here, ultrastructural analysis of synapses establishes that prodromal AD mice have reduced synaptic vesicle content within readily releasable and reserve pools. This new finding is consistent with readouts of presynaptic activity such as increased post-tetanic PPF ratios observed in these AD mice. PPF, a form of presynaptic Ca^2+^-dependent short-term plasticity, reflects the probability of neurotransmitter vesicle release, with increased ratios signifying a reduced release probability. The reduction in vesicle pools manifests as decreased PTP and synaptic strength at the single cell as well as local circuit levels in the AD mice. It has previously been shown that post-tetanic potentiation (PTP) correlates well with recruitment of synaptic vesicles from the reserve pool, thus PTP defects can be related to deficiencies in this specific population of vesicles [104, 105]. Interestingly, the opposite pattern is described in a mouse model of Fragile X syndrome. Here, an enlargement of the ready releasable and reserve pools with enhanced vesicle recycling results in increased STP and synaptic excitability [106]. Each of these scenarios results in impaired cognitive functions, suggesting that precise regulation of vesicle dynamics is required for proper learning and memory processing.

Synapses with high release probability, such as the hippocampal Schaffer collateral synapse described in the 3xTg-AD and other AD mouse models, show greater depletion of synaptic vesicles [107]. Vesicle release probability (p) is inversely associated with PTP expression, and increases in (p) are associated with decreased PPF ratios [55, 67–69]. This is consistent with the Ca^2+^-mediated increase in release probability, depleted presynaptic stores, and blunted PTP and network propagation described in the 3xTg-AD mice. Notably, synaptic strength is dependent upon the 3rd-4th power of [Ca^2+^] [108], and this steep power function is a critical variable influencing plasticity defects in AD mice where synaptically-evoked Ca^2+^ levels within spines are 2–3 times higher than in controls [21, 103]. Under these extreme conditions, the markedly increased Ca^2+^ does not translate into stronger synaptic functions, but rather, creates a deficient or overtaxed synaptic environment. Furthermore, the elevated Ca^2+^ is positioned to drive structural deficits within spines, and destabilize pre-postsynaptic integrity. The structural integrity of synapses, defined operationally as a stable pre-postsynaptic alignment through proteins such as adhesion molecules, is necessary for effective neurotransmission and synaptic function. A subclass of these adhesion molecules include the cadherins, a group of Ca^2+^-dependent glycoproteins which link the actin filament network associated with synaptic membranes. N-cadherin contributes to the structural and functional organization of the synaptic complex by ensuring this adhesion and close junction of pre and postsynaptic elements necessary for efficient synaptic communication and plasticity [81]. Thus, a Ca^2+^-mediated disruption in cadherin function would further disrupt synaptic stability, in addition to the well-described loss of mushroom spines through actin destabilization [2].

Reduced vesicle stores and synaptic depression at the single neuron level can contribute to broader hippocampal network defects. VSD imaging has previously been used to show that abnormal excitability may be an important contributor to early-stage AD pathophysiology [99]. To our knowledge, the current study is the first use of VSD imaging to examine how the hippocampal CA1 network is affected in AD. In the 3xTg-AD mice, we expected that the increased RyR-evoked Ca^2+^ would lead to enhanced Ca^2+^-activated K^+^ channel activation [28] and thus decreased neuronal excitability manifesting as spatially and temporally blunted CA1 depolarization. Our results revealed that network plasticity differences between NonTg and 3xTg-AD mice at this age were restricted to a surprising location: the SO subfield of CA1. The SO is largely comprised of the basilar dendrites of the CA1 pyramidal cells, and receives a strong input from CA2. This local circuit is important for social learning [109, 110], which is relevant in light of recent studies showing a link between increased social activity and increased resilience to developing AD [111, 112]. The SO also contains an important class of interneurons (oriens lacunosum-moleculare or OLM cells), which coordinate cell assemblies and cross frequency coupling, and drive theta oscillations and gate LTP [110]. Thus broader effects on memory encoding may also manifest as a result of CA2-SO network defects. Indeed, the impaired network plasticity in the SO of 3 month-old 3xTg-AD mice may contribute to the working memory deficits present at this age [11, 12]. The CA2-SO circuit is also associated with driving socially-related behaviors and learning. In several mouse models of AD, social learning and responses are impaired at an early stage, often prior to overt spatial memory defects [113–117] suggesting that overt disruptions in the CA2-SO circuit precede those within the CA3-CA1 SR Schaffer collateral circuit in AD. This may reflect early selective changes within the CA2-SO circuit which suppress signal propagation and PTP [118] through this hippocampal subfield, such as increased expression of inhibitory GABA Aα2, α5, [119] and K_ATP_ channel subunits, as well as GFAP [120]. In addition to upregulated inhibitory mediators within the SO, alternative compensatory mechanisms may be recruited concurrently within the CA3-CA1 SR synapse which augment synaptic signaling, such as increased nNOS and NO [29]. Notably, the CA3-CA1 SR circuit appears relatively functional in 3xTg-AD mice at 3–4 months of age, as does LTP expression and the behaviors it supports such as spatial learning performance [6, 45]. Future studies will examine dendritic Ca^2+^ signaling and synaptic plasticity in the SO of CA1 in detail, with the expectation that significant plasticity deficits will be observed in AD mice at this age, thus exposing a localized vulnerability within hippocampal subfields.

Regarding the use of the 3xTg-AD mouse model, concerns have been expressed about the consistency of the pathological phenotype across colonies, with delays in the amyloid and tau phenotype anecdotally reported. While we are aware of these issues, we have measured consistent responses for over a decade in this mouse model which may reflect the knock-in of the human mutant presenilin gene (which replaces the murine presenilin 1 gene, and is thus expressed under the endogenous murine promoter) rather than the transgenic expression of mutant tau and APP under exogenous promotors such as Thy1 [17, 29]; current study. As a matter of scientific practice, it is important to validate findings across mouse models to confirm that underlying mechanisms are representative of a pathological feature and not an artifact within a single mouse model. Thus, to extend our findings beyond the 3xTg-AD model, we have replicated the fundamental experimental findings and/or identified existing comparable studies using mouse models expressing different complements of AD-associated transgenes or knock-in approaches. For example, in the TgCRND8 model (APP KM670/671NL + V717F), we demonstrate similar patterns of upregulated RyR-evoked Ca^2+^ release and spike-evoked Ca^2+^ responses in hippocampal neurons as the 3xTg-AD mouse, and a marked suppression of PTP and E-LTP in the PS1/APP (APP_SWE_/PS1_M146V_) models (Additional file 1: Figure S1). The pathogenic ER Ca^2+^ dyshomeostasis component of AD has been consistently demonstrated over many years across numerous AD mouse models including the PS1_M146V_KI [17, 111], PS1/APP (APP_SWE_/PS1_M146V_) [112], PS1/APP (C57BL/6-SJLF1-APP+/−/C57BL/6-D2F1-PS1+/−; 113) and Tg2576 [114] among others [15, 22]. Furthermore, human cells obtained from AD patients demonstrate similar Ca^2+^ signaling abnormalities [20] and RyR dyshomeostasis is identified in non-human primates with AD-like pathology [115]. Reversal of calcium dysregulation and related AD pathology with RyR negative allosteric modulators has been demonstrated in two PS1/APP lines (APP_SWE_/PS1_M146V_, 59; C57BL/6-SJLF1-APP+/−/C57BL/6-D2F1-PS1+/−; 113), Tg2576 [114], and PS1_M146V_KI mice [17]; and the restoration of normal Ca^2+^ signaling results in reduced amyloid and tau pathology, normalized synaptic transmission and plasticity, and improved cognitive function [59, 113, 114, 116, 117]. Additional pathogenic Ca^2+^-mediated features that are well-characterized in 3xTg-AD mice are also detailed in other AD mouse models. For example, increased Ca^2+^-activated K^+^ channel activity driven by upregulated RyR-Ca^2+^ [28] is verified in the TgCRND8 model along with its effects on neurotransmission and neuronal excitability [118, 119]. The increase in the frequency of spontaneous neurotransmitter vesicle release at the CA3-CA1 synapse has been replicated in two additional lines of AD mice [59, 113]. The increased nNOS in 3xTg-AD mice, driven by RyR-evoked calcium release [29], is similarly upregulated in the TgCRND8 model [120]. Thus, upon this background of consistently reported ER Ca^2+^ dysregulation, increased frequency of Ca^2+^-dependent vesicle release, synaptic depression, and altered membrane properties identified in several mouse models of AD across time, as well as human cells and primates, we feel there is low risk in attributing our findings to a mouse-line specific artifact. It is more probable that the synaptic and physiological deficits we report are a pathological consequence of altered Ca^2+^ homeostasis that is integral to the AD disease process.

## Conclusions

In summary, we reveal a sequence of novel synaptic defects in early-stage AD mice that have far-reaching implications for synaptic plasticity encoding and memory formation. Particularly significant is the reduction in active neurotransmitter vesicle stores in CA3 terminals that is associated with STP defects. We show the CA1 SO subfield is particularly vulnerable, thus bringing attention to a hippocampal circuit associated with social memory. Abnormally high synaptically-generated Ca^2+^ in dendritic spines generated during the tetanus and further increased through STP stages suggests a form of maladaptive meta-plasticity impacting functions central to memory formation and stabilization. These early structural and functional synaptic defects are likely linked to core mechanisms of memory loss in later AD stages.

## Additional file

Additional file 1: **Figure S1.** Additional AD mouse models demonstrate abnormal RyR-specific Ca^2+^ release and suppressed STP. (A) Bar graph shows peak evoked Ca^2+^ responses from TgCRND8 +/− mice (gray) and litter mate controls (black) from either RyR-sensitive ER stores (left, t (1,8)=2.37; *p* < 0.05), or from voltage-gated Ca^2+^ channels activated by a train of action potentials (right, *p* > 0.05). * = significantly different from littermate control. (B) Short-term plasticity such as PTP and E-LTP are disrupted in the APP_SWE_/PS1_M146V_ mouse model (gray squares, *n* = 5), as it is in the 3xTg-AD (black circles, *n* = 6), compared to background strain NonTg controls. Averaged PTP and E-LTP fEPSP slope values from controls are shown with a dashed line over those epochs to allow for visual clarity. **Figure S2.** Relative increases in synaptically-evoked Ca^2+^ responses during plasticity epochs. (A). Dendritic Ca^2+^ responses during the tetanus, PTP, and E-LTP phases indicated as the % increase over the 30 Hz baseline response for each mouse model. These percentages were calculated from the raw data responses as shown in Fig. 4. (B). From the same raw data series, the bar graphs show the fold-increase in the synaptically-evoked Ca^2+^ response from the 3xTg-AD mice relative to the response from the NonTg response during each epoch (baseline, tetanus, PTP, E-LTP). (PDF 305 kb)

## Abbreviations

3xTg-AD: triple transgenic AD mouse model
aCSF: artificial cerebrospinal fluid
AD: Alzheimer’s disease
AP: action potential
APP: amyloid precursor protein
CA1: Cornu Ammonis area 1
CA2: Cornu Ammonis area 2
Ca^2+^: calcium
CA3: Cornu Ammonis area 3
CICR: calcium-induced calcium release
CMOS: Complementary metal–oxide–semiconductor
E-LTP: early long term potentiation
EM: electron microscopy
EPSP: excitatory post-synaptic potential
ER: endoplasmic reticulum
FAD: familial Alzheimer’s disease
fEPSP: field excitatory post-synaptic potential
IP_3_R: inositol triphosphate
IR-DIC: infrared differential interference contrast
ISI: interspike interval
LFP: local field potential
LTP: long term potentiation
LY: Lucifer Yellow
NonTg: non transgenic
PBS: phosphate-buffered saline
PCL: pyramidal cell layer
PPF: paired pulse facilitation
PPR: paired pulse ratio
PS: presenilin
PS-1: presenilin-1
PSD: post-synaptic density
PTP: post-tetanic potentiation
RyR: ryanodine receptor
Ryx: Ryanodex
SCS: sucrose cutting solution
sEPSP: spontaneous excitatory post-synaptic potential
SO: stratum oriens
SP: stratum pyramidale
SR: stratum radiatum
STP: short term plasticity
Tau: tau protein
VSD: voltage-sensitive dye
