# Supplementary figures and images for: Reduced presynaptic vesicle stores mediate cellular and network plasticity defects in an early-stage mouse model of Alzheimer’s disease
Source: Mol Neurodegener. 2019 Jan 22;14:7. doi: 10.1186/s13024-019-0307-7 (PMC6343260; doi:10.1186/s13024-019-0307-7)

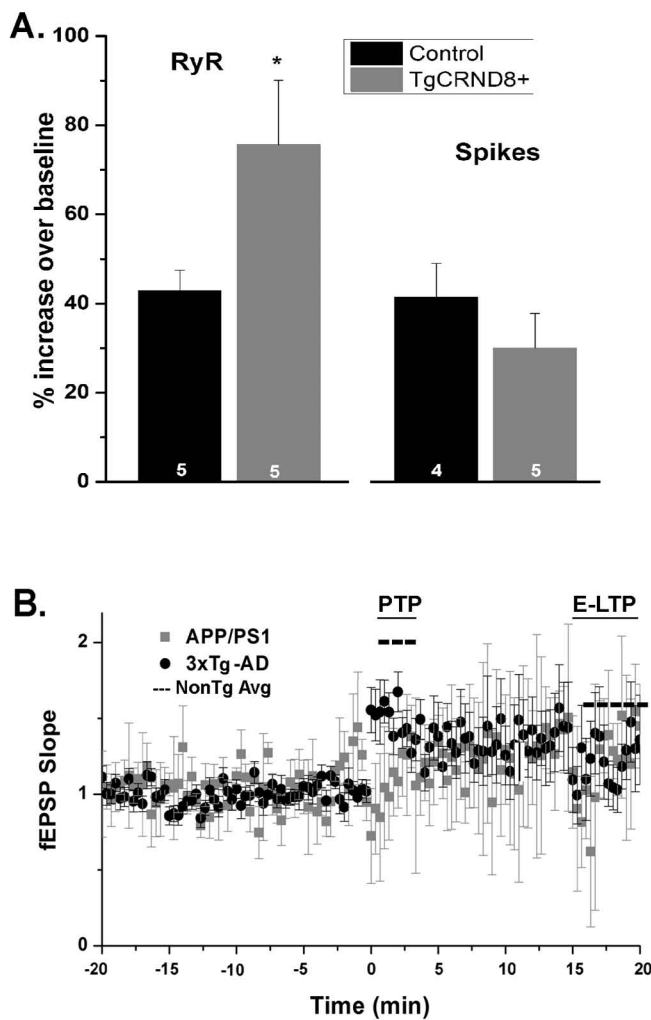

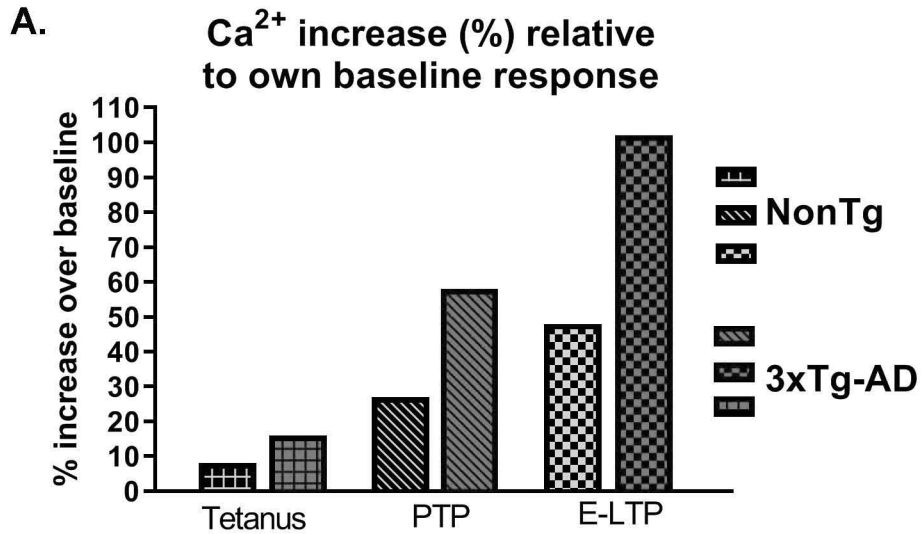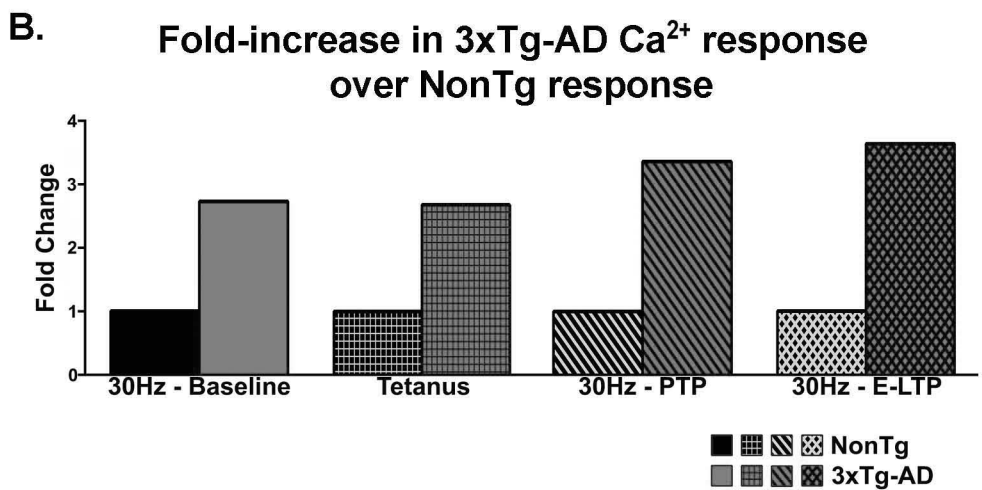

Supplement: Supplementary file 1 — Figure S1. Additional AD mouse models demonstrate abnormal RyR-specific Ca2+ release and suppressed STP. (A) Bar graph shows peak evoked Ca2+ responses from TgCRND8 +/− mice (gray) and litter mate controls (black) from either RyR-sensitive ER stores (left, t (1,8)=2.37; p < 0.05), or from voltage-gated Ca2+ channels activated by a train of action potentials (right, p > 0.05). * = significantly different from littermate control. (B) Short-term plasticity such as PTP and E-LTP are disrupted in the APPSWE/PS1M146V mouse model (gray squares, n = 5), as it is in the 3xTg-AD (black circles, n = 6), compared to background strain NonTg controls. Averaged PTP and E-LTP fEPSP slope values from controls are shown with a dashed line over those epochs to allow for visual clarity. Figure S2. Relative increases in synaptically-evoked Ca2+ responses during plasticity epochs. (A). Dendritic Ca2+ responses during the tetanus, PTP, and E-LTP phases indicated as the % increase over the 30 Hz baseline response for each mouse model. These percentages were calculated from the raw data responses as shown in Fig. 4. (B). From the same raw data series, the bar graphs show the fold-increase in the synaptically-evoked Ca2+ response from the 3xTg-AD mice relative to the response from the NonTg response during each epoch (baseline, tetanus, PTP, E-LTP). (PDF 305 kb) [file 13024_2019_307_MOESM1_ESM.pdf]
